# Parental attitudes and perceptions towards the recruitment of children to clinical trials: a cross-sectional survey

**DOI:** 10.3389/fped.2024.1490274

**Published:** 2024-11-13

**Authors:** Ibrahim Farhan Safra, Shaikha Jabor Alnaimi, Gehad Gad, Aliamma Abraham, Ahmad Hassan Al-Hammadi, Mohammad A. A. Bayoumi, Fawziya Alyafai, Ashraf Gad

**Affiliations:** ^1^ Neonatal Intensive Care Unit, Women’s Wellness and Research Center, Hamad Medical Corporation, Doha, Qatar; ^2^ Pharmacy Department, Women’s Wellness and Research Center, Hamad Medical Corporation, Doha, Qatar; ^3^ Data Science Department, Brooklyn College, Brooklyn, NY, United States; ^4^ General Pediatrics Department, Sidra Medicine, Doha, Qatar; ^5^ Pediatric Endocrinology Department, Sidra Medicine, Doha, Qatar

**Keywords:** neonatology, pediatrics, clinical trials, parental attitude, cross-sectional survey, child recruitment, parental consent, parental perceptions

## Abstract

**Background:**

Clinical trials (CTs) in children are critical for understanding and treating childhood diseases. However, there trials require prior permission from parents. We evaluated parental attitudes and perceptions regarding the recruitment of their children in CTs.

**Methods:**

We used a cross-sectional survey questionnaire targeting parents of children admitted to the neonatal and pediatric departments in two tertiary hospitals in Qatar. The survey was administered by investigators and was composed of two domains to assess the knowledge and attitude of parents regarding children's enrollment in CTs, in addition to the participant's demographics domain.

**Results:**

Of the 167 questionnaires offered to parents, we received a total of 138 responses, resulting in a response rate of 82.6%, with the majority being women (72%). Many parents (75%) expressed willingness to enroll their children in CTs. However, 66% opposed new experimental treatments for their child, while 41% agreed to new treatments if they had previously been used in the medical field. Logistic regression analysis revealed key predictors influencing parents’ decisions to include their children in CTs, including having a newborn (aOR = 17.651, *p* < 0.001), families with five or more members (aOR = 3.293, *p* = 0.012), collecting blood samples (aOR = 8.602, *p* = 0.003), performing additional tests on collected samples (aOR = 4.115, *p* = 0.046), belief in helping others (aOR = 8.744, *p* = 0.002), and the option of home therapy (aOR = 7.090, *p* = 0.004).

**Conclusion:**

Many parents are open to enrolling their children in CTs, particularly when treatments have been previously used. Factors like having a newborn, large family size, blood collection, additional tests, and home therapy influence their decisions. Clear communication can enhance recruitment in pediatric trials.

## Introduction

The world has witnessed a remarkable 60 percent reduction in under-five mortality, from 93 deaths per 1,000 live births in 1990 to 38 deaths per 1,000 live births in 2019. However, the worldwide burden of child and adolescent mortality remains significantly high (1). In 2019, 7.4 million children, adolescents, and teenagers (0–14 years) died, mostly from preventable or treatable diseases. Additionally, the burden of child mortality remains unequally allocated. Developing countries have the highest under-five death rate (74 per 1,000 live births), which is almost nine times that of developed countries (1). Despite these observations, children constitute about 27% of the global population, and as of December 2019, only 11,738 pediatric clinical trials (CTs) were registered (2).

Due to the scarcity of pediatric studies, doctors often extrapolate adult data when prescribing medications to children and newborns. Children are not “little adults” but a heterogeneous group that ranges from preterm newborns to post-pubertal teenagers (3, 4). The clinical presentation of their diseases may significantly differ from adults, and they may also be suffering from child-specific diseases. It is crucial to remember that children's physiology, development, psychology and pharmacokinetics significantly differ from that of adults, and these differences exist from birth until adolescence (5, 6). Failure to address these differences may lead to suboptimal treatment, unanticipated responses, severe drug reactions, and toxicity that can impair development and future generative capability.

Results from randomized controlled trials (RCTs) provide the highest level of evidence when evaluating clinical care recommendations (7). However, conducting RCTs is a challenge due to the low participation rates (3%–20%), long recruitment periods (up to years) and financial cost (4%–16% of the research budget). Generally, children are considered more challenging to recruit in CTs than adults. This is due to the inability of children to provide informed consent and the need to receive permission from parents while preserving the child's autonomy (6). Moreover, recent studies indicate that many physicians are hesitant to ask families to engage in research, which may affect their referral behaviour, possibly due to unfamiliarity with the study design (8, 9). Paediatricians may fail to invite eligible participants due to ethical concerns or expected subject rejection. Nevertheless, paediatricians’ involvement in the recruitment process is critical since the willingness of families and children to engage in clinical research is highly influenced by their treating physician's recommendation (9).

When parents are approached to consider their child's enrollment in a trial, their main concern is their child's care. This role is very distinct from an adult choosing their involvement in a trial. While grappling with complicated medical facts is an inherent part of trial decision-making, when the decision is made on behalf of a dependent child, however, it becomes potentially overwhelming (10). Despite this fundamental distinction between adult and pediatric medical trials, very little is known about how the unique role of parents affects the child's enrollment and trial execution. Therefore, we aimed to explore the parents’ role in terms of attitude and perception in enrolling their children in CTs.

## Patients and methods

### Study population and duration

We aimed to include parents of newly born infants and children admitted to the pediatric wards in two tertiary hospitals in Qatar. The study was conducted from September 1st, 2013 to March 1st, 2014.

### Study instrument and data collection

The questions were developed by the research team and were reviewed for face validity by a panel of neonatologists and pediatric physicians. The questionnaire, broadly, was comprised of two domains. The first domain included demographic characteristics, while the second domain included study questions or variables. The demographic comprised two sub-domains of information one about the parent and the other about the child. Also, the study questions were composed of two sub-domains. The first was related to parental attitudes towards enrolling their children in CTs and the second included the items about the perception of the parents regarding enrolling their children in CTs.

The questionnaire was administered in person by the investigators to parents of stable term newborns (gestational age ≥37 weeks) in the postnatal ward, as well as to parents of term newborns admitted to the neonatal intensive care unit (NICU) or intermediate care, and to parents of children admitted to the pediatric ward or short-stay unit. Parents of preterm and sick and unstable babies in the NICU were excluded from enrollment in the study due to the complexity of the decision-making process. The study participants were treated as per the declaration of Helsinki (11). The study was approved by the medical research centre (12052/12). Informed consent was taken from each study participant before survey administration. The informed consent stated the nature of the study, confidentiality of the participant's information and willingness to participate.

### Statistical analysis

Descriptive statistics, including frequency, percentage, mean, and standard deviation (SD), were used to evaluate the sample demographics and parents’ attitudes and perceptions regarding the enrollment of their child in the CTs as appropriate. Univariate and multivariate regression analyses were conducted to identify significant predictors influencing parents’ decisions regarding their children's participation in CTs. Factors with *p* < 0.1 in the univariate analysis were considered for inclusion in the multivariate analysis. Odds ratios (OR) and 95% confidence intervals (CI) of odds ratios were obtained. The statistical package for social sciences (SPSS v27, IBM Corp. Armonk, NY) and Jamovi Version 3.1 were used for statistical analysis.

## Results

### Study participants

A total of 167 questionnaires were offered to parents, and we collected a total of 139 responses. One response was excluded due to being incomplete and the child's discharge from care. The remaining 138 responses were fully completed, resulting in a response rate of 82.6%.

### Demographics

Table 1 presents the baseline characteristics of the parents. The mean age of participants was 32.0 ± 6.33 years, with the majority being female (72%). The highest level of education among parents was graduate (37%), followed by college education (29%). Most parents were expatriates (85.5%), while 14.5% were Qatari nationals. In terms of regional background, the majority were from the Middle East and North Africa (73.1%), followed by Asians (16.7%) and Africans (6.6%). Nearly half of the parents were full-time employees (45%). Most families lived in apartments (57%), compared to houses (29%) or shared accommodations (14%). The majority of families (62%) had fewer than five members, and most lived in either 3–4 rooms (42%) or 1–2 rooms (40%).

**Table 1:** Sociodemographic characteristics of parents.

Variable	Category	*N* (total = 138)	%
Age (years), mean ± SD	32.0 ± 6.33		
Gender	Male	39	28.0
	Female	99	72.0
Ethnicity	Middle Eastern/North African	101	73.1
	Asian	23	16.7
	African	9	6.6
	Other	5	3.6
Education	College	40	29.0
	Graduate	51	37.0
	Secondary school	35	25.0
	None	12	9.0
Employment	Full time	71	52.0
	Part-time	7	5.0
	Unemployed	60	43.0
Housing	House	40	29.0
	Apartment	78	57.0
	Sharing	20	14.0
Family members	<5	86	62.3
	≥5	52	37.7
Number of rooms at home	1–2 rooms	56	40.0
	3–4 Rooms	58	42.0
	5–6 Rooms	12	9.0
	7–8 Rooms	9	7.0
	8–10+	7	6.0

SD, standard deviation.

Table 2 summarizes the characteristics of the children of surveyed parents. Data were collected for a total of 138 children, with a mean age of 4.76 ± 10.34 days for newborns and 32.17 ± 42.50 months for older children. About 30% were first-born, 37% were second-born, and 21.8% were third or fourth-born. Sixty-one per cent were admitted to the postnatal ward, followed by 16% in the intermediate care nursery. Sick newborns and pediatric patients represented 23% of the children, with 16% in the pediatric ward, 4% in the NICU, and 3% in the short-stay unit.

**Table 2:** Demographic characteristics of children.

Variable	Category	*N* (total = 138)	%
Gender	Male	68	49.0
	Female	70	51.0
Hospital unit/ward	Postnatal (newborn) ward	84	61.0
	Intermediate care	22	16.0
	Intensive care	6	4.0
	Pediatric ward	22	16.0
	Short stay unit	4	3.0
Birth order	1st	42	30.4
	2nd	51	37.0
	3rd	15	10.9
	4th	15	10.9
	5th	6	4.3
	6th–10th	9	6.5
Age of child (mean ± standard deviation)	Newborn, days (*n* = 111)	4.76 ± 10.34	
	Child, months (*n* = 27)	32.17 ± 42.50	

### Parental attitudes towards participation in RCTs

**Figure 1:**
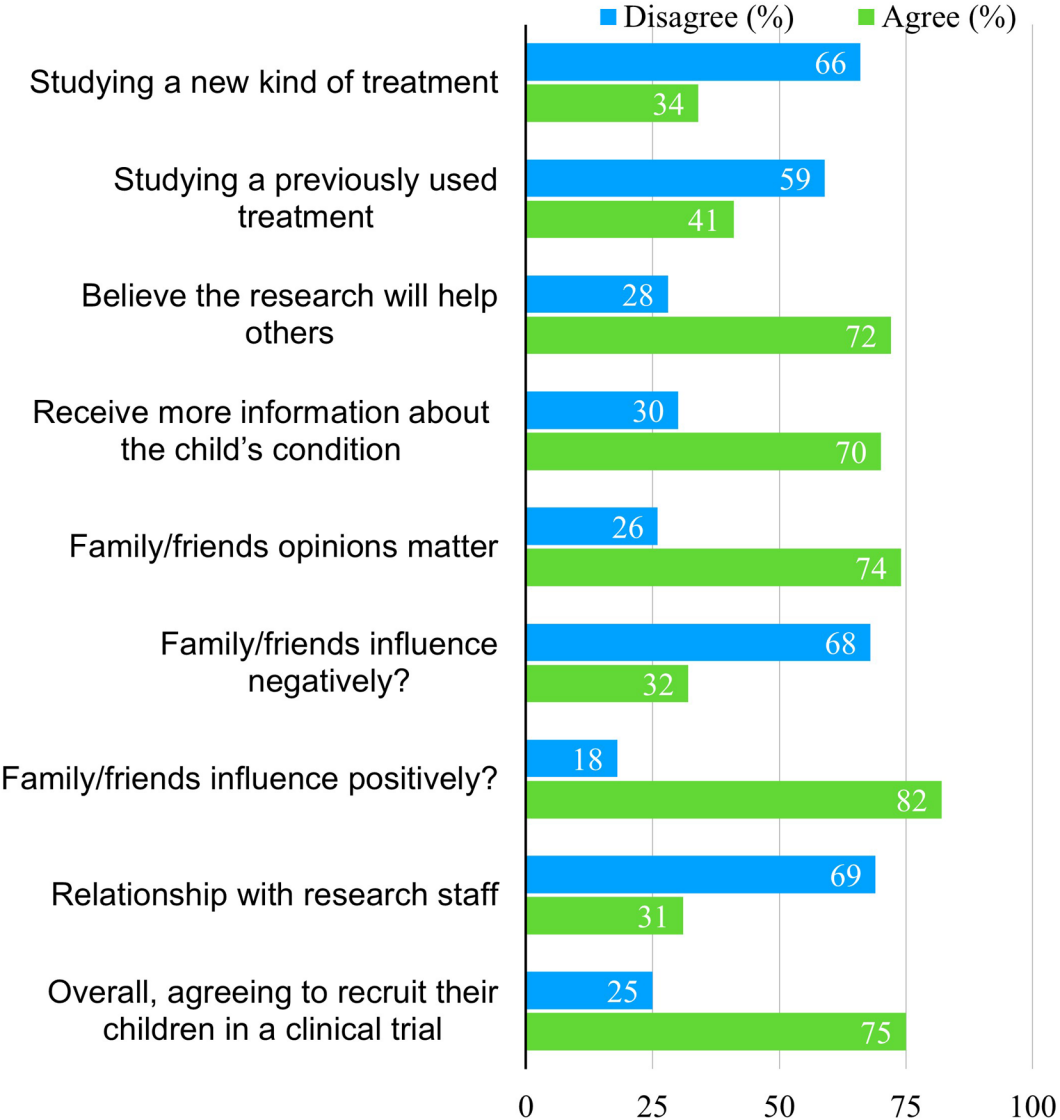
Parents’ attitudes toward recruiting their children in a trial (*n*).

Parental attitudes toward enrolling their children in CTs are shown in Figure 1. Most parents (66%) disagreed with involving their children in trials that introduce new treatment methods, including new drugs, techniques, or machines, while 59% disagreed if the treatment had been previously used. However, 72% of parents agreed to participate if they believed the research would help others, and 70% agreed if they had received more information about their child's condition.

Most parents (74%) felt that the opinions of family and friends mattered, and 82% said their influence was positive, compared to 32% who believed it would negatively affect the decision. Only 31% agreed that a relationship with research staff would influence their decision to volunteer their children for CTs. Overall, 75% of parents expressed willingness to enroll their children for future CTs.

**Figure 2:**
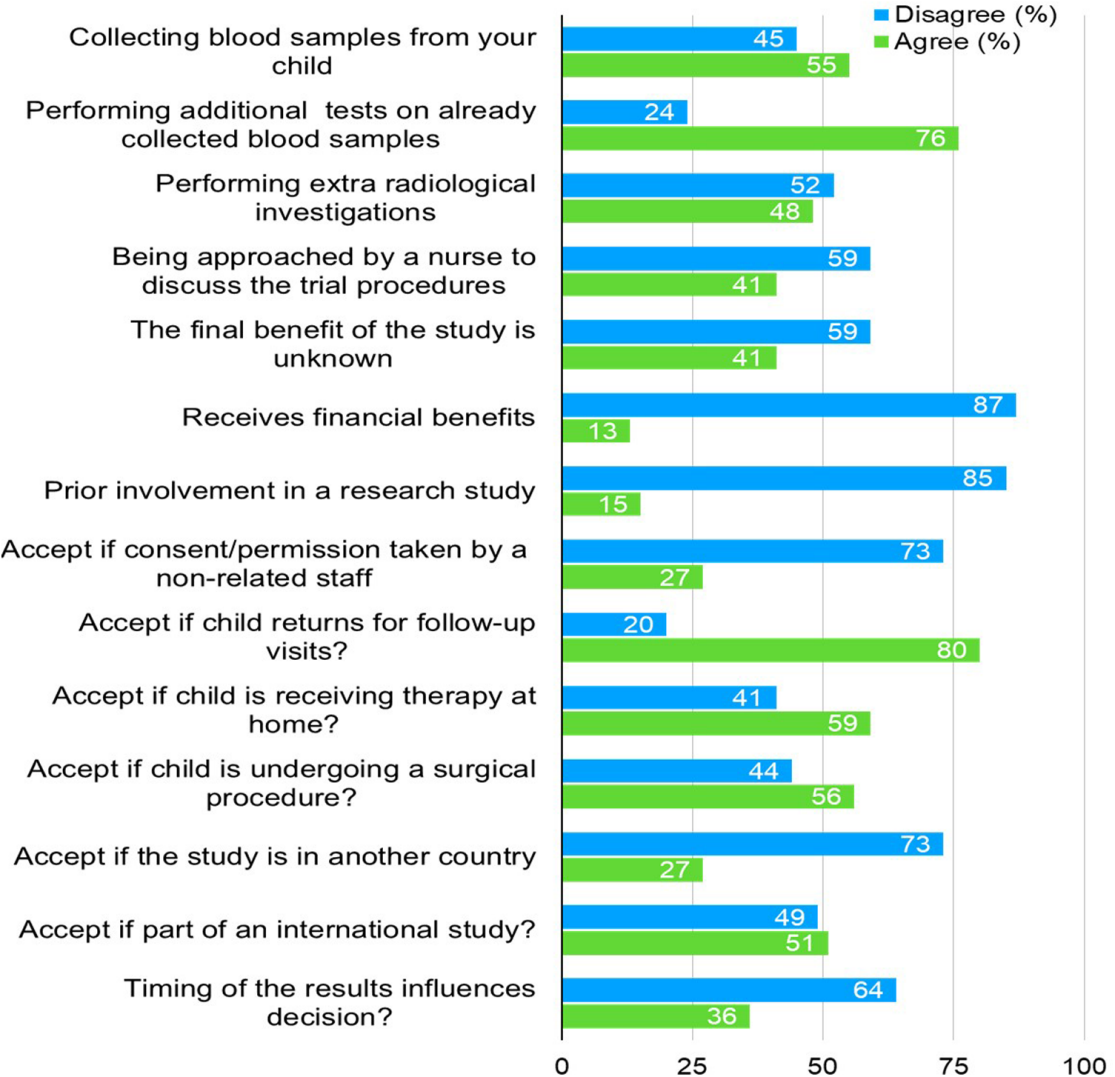
Factors affecting parents’ decision toward recruiting their children in a trial.

Figure 2 summarizes factors affecting parents’ decisions regarding enrolling their children for CTs. A majority (55%) agreed to collect blood samples, and 76% supported performing additional tests on already collected blood samples. However, 52% disagreed with extra radiological investigations, while 59% disagreed when approached by a nurse for the trial proceedings. Most parents (87%) disagreed with receiving financial benefits (i.e., financial benefits, gifts, free medications and free visits), and 85% had not been involved in previous research. Despite this, 80% were open to their child returning for follow-up visits, and 59% were comfortable with home therapy. However, only 27% agreed if the study was in another country, and 51% accepted trials as part of an international study. Lastly, 64% disagreed that the timing of the results would influence their decision.

### Univariate and multivariate regression analysis

Table 3 summarizes the results of univariate regression analyses highlighting several significant factors influencing parents’ decisions regarding their children's participation in CTs. Of these, five factors remained significant predictors for the decision-making in the multivariate model. Parents of newborns were significantly more likely to agree to participation, with an adjusted odds ratio (aOR) of 17.651 (95% CI: 4.178–74.574, *p* < 0.001). Families with five or more members were also more likely to agree (aOR = 3.293, 95% CI: 1.298–8.356, *p* = 0.012). Collecting blood samples from the child (aOR = 8.602, 95% CI: 2.117–34.954, *p* = 0.003) and performing additional tests on already collected samples (aOR = 4.115, 95% CI: 1.027–16.488, *p* = 0.046) were positively associated with participation. Parents who believed the research would help others were more likely to agree (aOR = 8.744, 95% CI: 2.197–34.800, *p* = 0.002), and those whose children would receive therapy at home had a higher likelihood of agreeing (aOR = 7.090, 95% CI: 1.895–26.522, *p* = 0.004).

**Table 3:** Regression analysis of predictors for parental agreement to enroll their children in clinical trials.

Variable/predictor	Univariate^a^			Multivariate^b^		
	Odds ratio	95% CI	*P*-value	Adjusted odds ratio	95% C.I.	*P*-value
Nationality, expatriates^c^	4.787	1.882–12.863	0.002			
Research location-intermediate nursery/postnatal ward^d^	8.221	3.398–19.889	<0.001			
The child is being a newborn	18.286	6.606–50.624	<0.001	17.651	4.178–74.574	<0.001
Family members, number ≥5	1.715	0.986–2.983	0.056	3.293	1.298–8.356	0.012
The family lives in a house^e^	0.361	0.161–0.808	0.013			
Studying a new kind of treatment	2.540	1.014–6.361	0.047			
Studying a previously used treatment	3.774	1.513–9.412	0.004			
Collecting blood samples from the child	10.253	3.880–27.098	<0.001	8.602	2.117–34.954	0.003
Performing additional tests on already collected samples	7.549	31.57–18.049	<0.001	4.115	1.027–16.488	0.046
Performing extra radiological investigations	5.156	2.064–12.875	<0.001			
Being approached by a nurse	2.425	1.035–5.681	0.041			
The final benefit of the study is unknown	7.902	2.603–23.992	<0.001			
Given more information about the child's condition	2.221	0994–4.961	0.052			
Parents believe the research will help others	2.194	0.970–4.963	0.059	8.744	2.197–34.800	0.002
Consent is taken by a non-related staff	5.252	1.502–18.394	0.009			
The child will be receiving therapy at home	6.710	2.819–15.969	<0.001	7.090	1.895–26.522	0.004
If the child is undergoing a surgical procedure	3.892	1.715–8.831	0.001			
If the trial is part of an international study	2.470	1.111–5.494	0.027			

The reference group is other hospital units: “Neonatal Intensive Care Unit, Pediatric Ward, and Short Stay Unit”.

^a^ The reference group for questionnaire variables is “No”.

^b^ Stepwise forward LR multivariate regression analysis.

^c^ Reference group is “Qatari Nationals”.

^d^ Reference group is apartment and sharing.

## Discussion

The participation of children in CTs is a complex dilemma. Parents play a vital role in enrolling children to CTs as they are ultimately responsible for accepting or denying the child's participation. Studies show patients’ participation in research increases the recognition of their disease and positively influences overall patient management (12).

In the current study, parents demonstrated a positive attitude towards enrolling their children in CTs. Our data showed that extra benefits like financial compensation, gifts, free medications, or even complimentary visits could not sway parents’ decisions about their child's enrollment. The results also showed that third-party permission, living status, relationship with research staff and timing of results did not affect the parents’ decision.

Comparable to our results, a study from the United States investigating barriers and facilitators to CT participation among parents of children reported an overall positive parental attitude (13). Parent's negative perspective on enrolling their child to a CT may be due to barriers such as a lack of information about the risk and trial procedures and the possibility of receiving a placebo. As reported in the current study, these barriers can be facilitated by improving researchers’ communication and ultimately parents’ understanding.

Unlike a previous study that has shown that financial incentives may affect parental decisions to participate in clinical studies (13), the decision of parents in our study was not affected by financial incentives. This is likely to differ among countries based on the socioeconomic status, cost of living per capita income and nature of the health care in Qatar. In Qatar, the government provides comprehensive national healthcare coverage where residents receive medical care, especially in government hospitals at little to no personal cost. The availability of free or subsidized healthcare reduces the financial burden on families, making financial incentives less impactful in their decision-making for CT participation. However, due to ethical considerations, the issue of financial compensation is complex, and there is significant variance across studies regarding the type of compensation given to participants (10, 14).

The results showed a higher number of mothers giving parental permission than fathers. Although high in percentage, the finding is consistent with similar studies (15–17), mothers tend to be the primary decision-makers for their children's healthcare. In the review by Fisher et al., it was highlighted that mothers are more likely than fathers to engage in discussions and consent for pediatric CTs due to their more frequent role as primary caregivers (16).

The study findings indicated that large families are likely to agree to children's enrollment in a CT. This openness to RCTs might be due to parents in larger families being more familiar with the healthcare systems, leading to a greater comfort level with clinical research. Additionally, parents in large families may perceive trial participation as an opportunity to access additional care or medical follow-ups, potentially benefiting all their children. The perception that a CT will help others, including other children, also strongly influences decision-making. Studies have found that altruism and the belief that participation in research could advance medical knowledge or benefit society are significant motivators for parents (17).

With regards to CT procedures, although most parents agreed to a trial involving performing a lab test, response rates dropped by one-quarter when the question involved blood collection, a similar finding to a study by Lee et al. that assessed parental willingness to enrol their children in surgical trials (18). Of interest, our study demonstrates that although 72% of parents were mothers, 56% agreed to a CT involving surgical procedures. This finding opposes the results of similar studies, where women care, providers, were less willing to enrol their children in research involving surgery or randomization to surgery vs. no surgery (18). Parents who were less likely to permit surgical trials had higher socioeconomic status, more stress, and lower levels of trust (19).

Some studies that assessed recruitment of paediatrics in research reported that one of the major barriers to recruitment was parents’ concerns about negatively affecting their child's health by exposing them to unknown treatments, that the investigators may not prioritize their child's health and that their child would be used as a “guinea pig” (20, 21). On the other hand, in a study that assessed the enrollment of neonates, 75% of parents indicated that they trusted their doctor and would accept participating in a trial if it was recommended by them (22). Therefore, it is likely that parents’ willingness to enrol their children may vary depending on the age of the child and the nature of the clinical condition. In addition, the participant-investigator relationship is likely to influence parents’ willingness to child enrollment, as trust in the investigator provides moral support to parents. Family dynamics and size may influence parental decision-making in general, as larger families could indicate more shared responsibility or different socio-economic pressures that affect participation in clinical studies.

### Study limitations

This study has several limitations. First, subjective bias is possible, as we did not cover all aspects of the parental perspective, particularly from parents of unstable newborns, which may not reflect the views of parents of critically ill infants. The attitudes and decision-making processes of these parents could differ, particularly regarding permission for experimental treatments. Second, we did not consider the investigators’ perspectives, which may significantly influence parents’ attitudes and willingness to volunteer their children for CTs.

Lastly, our sample population may differ significantly from those in other countries, potentially limiting the generalizability of our findings.

## Conclusions

The current study highlighted important factors regarding the enrollment of children in CTs. Overall, parents expressed a positive attitude toward enrolling their children, although many opposed new treatments. Certain predictive factors positively influenced parents’ decisions to children participate in CTs. These decisions can be further encouraged by improving parents’ understanding of CTs and fostering strong communication and relationships between parents and investigators. Future research should focus on examining how different types of CTs influence parental decision-making. Additionally, exploring the impact of family dynamics, child condition and parental roles could provide a deeper understanding of why parents agree to or decline participation in CTs for their children.

## Data Availability

The raw data supporting the conclusions of this article will be made available by the authors, without undue reservation.
